# Peristaltic Transport of Carreau-Yasuda Fluid in a Curved Channel with Slip Effects

**DOI:** 10.1371/journal.pone.0095070

**Published:** 2014-04-15

**Authors:** Tasawar Hayat, Fahad Munir Abbasi, Bashir Ahmad, Ahmed Alsaedi

**Affiliations:** 1 Department of Mathematics, Quaid-I-Azam University, Islamabad, Pakistan; 2 Nonlinear Analysis and Applied Mathematics (NAAM) Research Group, Faculty of Science, King Abdulaziz University, Jeddah, Saudi Arabia; University of Zurich, Switzerland

## Abstract

The wide occurrence of peristaltic pumping should not be surprising at all since it results physiologically from neuro-muscular properties of any tubular smooth muscle. Of special concern here is to predict the rheological effects on the peristaltic motion in a curved channel. Attention is focused to develop and simulate a nonlinear mathematical model for Carreau-Yasuda fluid. The progressive wave front of peristaltic flow is taken sinusoidal (expansion/contraction type). The governing problem is challenge since it has nonlinear differential equation and nonlinear boundary conditions even in the long wavelength and low Reynolds number regime. Numerical solutions for various flow quantities of interest are presented. Comparison for different flow situations is also made. Results of physical quantities are interpreted with particular emphasis to rheological characteristics.

## Introduction

The peristaltic pumping has prime importance for fluid transport from a region of lower to higher pressure. Peristaltic transport of fluids through different vessels of human physiological systems is known to physiologists as a natural mechanism of pumping materials. Such mechanism in fact is due to travelling contraction waves along a tube like structure. It is because of neuron-muscular properties of tubular smooth muscles in the physiological processes. In particular a peristaltic activity in biological systems is quite prevalent in the gastrointestinal, urinary, reproductive tracts, small blood vessels, intestines, lymphatic vessels and many other glandular ducts in a living body. Importance of peristaltic mechanism in the industrial applications is quite obvious for instance in sanitary and corrosive fluids transport, in roller, finger and hose pumps and blood pump in heart lung machine. This phenomenon for toxic liquid transport is employed in the nuclear industry. Having all such in mind much attention in the past has been given to the peristaltic flows in straight channels. In such investigations most biological fluids (such as blood, chyme) are treated as the viscous fluids (see [1]–[9]). In another study Mekheimer et al. [10] investigated the peristaltic motion of an incompressible viscous fluid due to asymmetric waves propagating on the horizontal sidewalls of a rectangular duct under long-wavelength and low-Reynolds number assumptions. Later some attempts have been made for peristaltic motion which take into account the viscoelastic properties of fluids. The viscoelastic fluids unlike the viscous liquid cannot be described by one constitutive relationship. Hence many models of non-Newtonian fluids have been proposed for their rheological characteristics. The governing equations in such fluids are also complicated, of higher order and more nonlinear than the Navier-Stokes equations. Few investigations in this direction may be represented by the refs [12]–[21]. Recently Abd elmaboud et al. [22] analyzed the heat transfer characteristics of a couple-stress fluid (CSF) in a two-dimensional asymmetric channel. Long wavelength approximation is used in the mathematical modelling in this analysis. Carreau-Yasuda (C-Y) model is amongst the models of non-Newtonian fluids which has advantage over the so-called power law fluid model. In fact the Carreau-Yasuda model contains five parameters to explain the fluid rheology when compared to two parameters in Power-law model. The shear thinning and shear thickening effect can be predicted by this model with large accuracy. It can predict the results of both Carreau and viscous fluid models in limiting situations. Gijsen et al. [23]–[24] investigated the non-Newtonian properties of blood in large arteries. In these studies authors compared the experimental results with the numerical data using C-Y model. A very good agreement is noticed. Andrade et al. [25] computed skin friction equation for turbulent flow in pipes using the C-Y model. Very recently Hayat et al. [26] examined the Hall and Ohmic heating effects on the peristalsis of C-Y fluid.

It is now known that curved channels in industrial and physiological processes are more realistic than the straight channels. Thus attention has been recently diverted to the peristaltic flows in the curved channels (see [27]–[31]). However no attempt is yet presented to examine the peristaltic transport of C-Y fluid in a curved channel. Such mathematical modelling thus is presented first time here. Problem formulation is completed by considering slip effects at the channel boundaries. Resulting nonlinear problem is solved numerically. Graphs are displayed and examined for the various parameters of interest.

## Problem Statement

Consider a channel of width 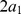 coiled in a circle with center 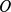 and radius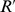. An incompressible fluid of Carreau-Yasuda (C-Y) type fills the channel. The axial and radial directions are denoted by 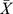 and 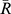 respectively. Flow in the channel is induced due to propagation of peristaltic waves travelling on the channel walls in the axial direction with constant speed 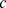. The geometry of peristaltic walls is given by the following relation:
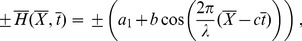
(1)where 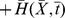 is the upper wall, 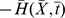 the lower wall, 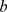 the wave amplitude, 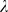 the wavelength and 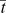 the time. Velocity field for such flow is 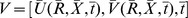. The conservation of mass and scalar components of momentum equation for two-dimensional incompressible flow are



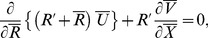
(2)




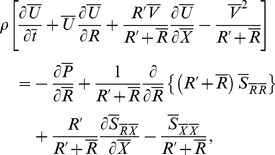
(3)

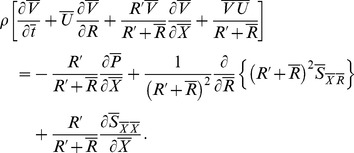
(4)


In above expressions 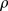 is the density of fluid, 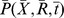 the pressure and 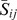 the components of extra stress tensor. It is obvious that the problem given through Eqs. (2–4) is unsteady in fixed (laboratory) frame. In order to make it steady we transform these equations in a frame of reference (wave frame) moving along the wave with the same speed. The transformations between two frames are
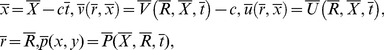
(5)in which 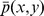 is the pressure and 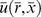, 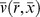 are the radial and axial components of velocity in the wave frame 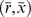 respectively. Relevant equations in wave frame become



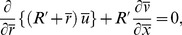
(6)




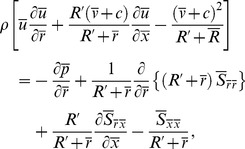
(7)




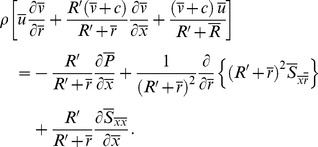
(8)


Making use of the following dimensionless quantities.
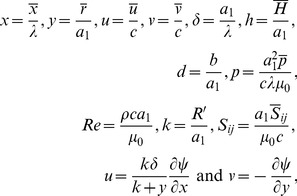
(9)equations (7) and (8) are reduced to



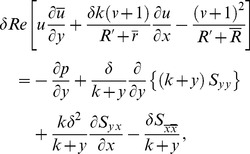
(10)




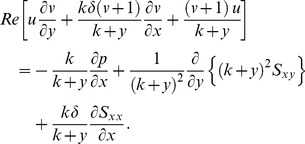
(11)and the continuity equation is identically satisfied. Here 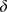 is the wavenumber, 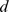 the amplitude ratio, 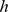 the dimensionless form of wall shape, 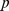 the dimensionless pressure, 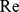 the Reynolds number, 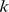 the curvature parameter and S*_ij_* the dimensionless components of the stress tensor. Further analysis is carried out under the assumption that width of the cannel is small compared to wavelength of peristaltic waves i.e. 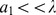 (or 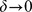). This assumption is usually called as long wavelength approximation. Such consideration is realistic when peristalsis for ureter, chyme movement in intestine and spermatozoa in ductus efferentes are considered. The Reynolds number is taken low. The long wavelength and low Reynolds number approximations are used extensively in the analysis of peristaltic flows (see refs. [2]–[4], [8]–[10]). It should be pointed out that the theory of long wavelength and zero Reynolds number remains applicable for case of chyme transport in small intestine [11]. In this case *c = 2* cm/min, *a_1_ = 1.25* cm and *λ = 8.01* cm. Here half width of intestine is small in comparison to wavelength. i.e. *a_1_/λ = 0.156*. The above equations in terms of such assumptions take the form



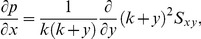
(12)




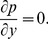
(13)


Eq. (13) states that 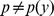. Eliminating pressure between Eqs. (12) and (13) we arrive at
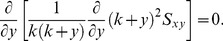
(14)


## Stress Tensor for Carreau-Yasuda Fluid

The extra stress tensor for Carreau-Yasuda fluid is given as [23]–[25]

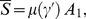
(15)where A_1_ is the first Rivlin-Erickson tensor and the apparent viscosity 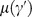 is defined as follows:



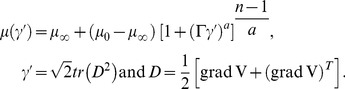
(16)


Here 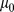 is the zero shear-rate viscosity, 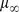 the infinite shear-rate viscosity and 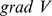 denotes the gradient of velocity vector. This particular fluid model has the tendency to define Newtonian fluid of viscosity 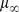 and 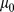 at the upper and lower ends of shear rate range whereas at intermediate values the shear thinning/thickening behavior is predicted through the parameters 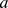, 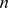 and 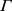 defining the transition conditions. Involvement of five parameters in this fluid model provides it preference over the so-called Power-Law model which contains two parameters to describe the rheology of fluid. As a limiting case the results of Carreau fluid model can also be retained by substituting 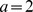. In wave frame we have
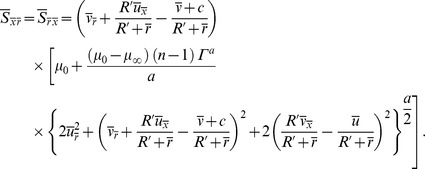



The dimensionless form of extra stress tensor under the low Reynolds number and long wavelength approximation becomes
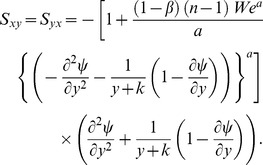
(17)


Here 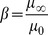 is the viscosity ratio parameter and 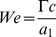 the Weissenberg number.

## Flow Rate and Boundary Conditions

Taking 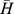 as function of 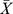 and 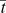, the dimensionless volume flow rate in laboratory frame is represented by the following expression
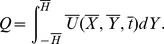



Above expression in wave frame is reduced to
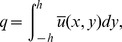
where 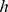 is function of 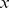 alone. The above two expressions yield



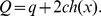



Time averaged flow over a period 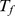 is defined by
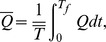
or



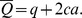



Defining 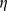 and 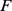 as the dimensionless mean flows in the laboratory and wave frames by
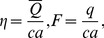
(18)then



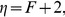
(19)with



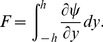
(20)


Nondimensional pressure rise per wavelength is computed by the relation
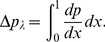
(21)


The subjected dimensionless boundary conditions are
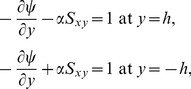
(22)where 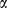 is the dimensionless velocity slip parameter and the dimensionless wall shape is given as follows:



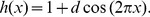
(23)


Substituting the value of 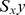 from Eq. (17) into Eqs. (14) and (22) we get
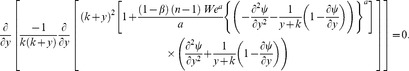
(24)




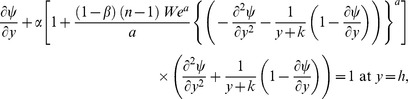
(25)

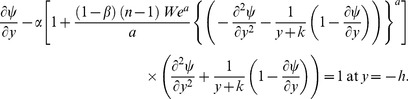
(26)


It should be pointed out that the results of Newtonian fluid can be recovered by substituting 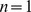 or 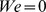. Also the case of planar channel is obtained for 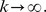 The above nonlinear equation subject to nonlinear boundary condition seems very difficult to solve analytically. Hence we compute the numerical solution by NDSolve of Mathematica. We have taken the step size equal to 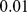 for variations in both 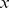 and 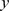. Obtained numerical results are analyzed graphically in the next section.

## Graphical Analysis

**Figure 1:**
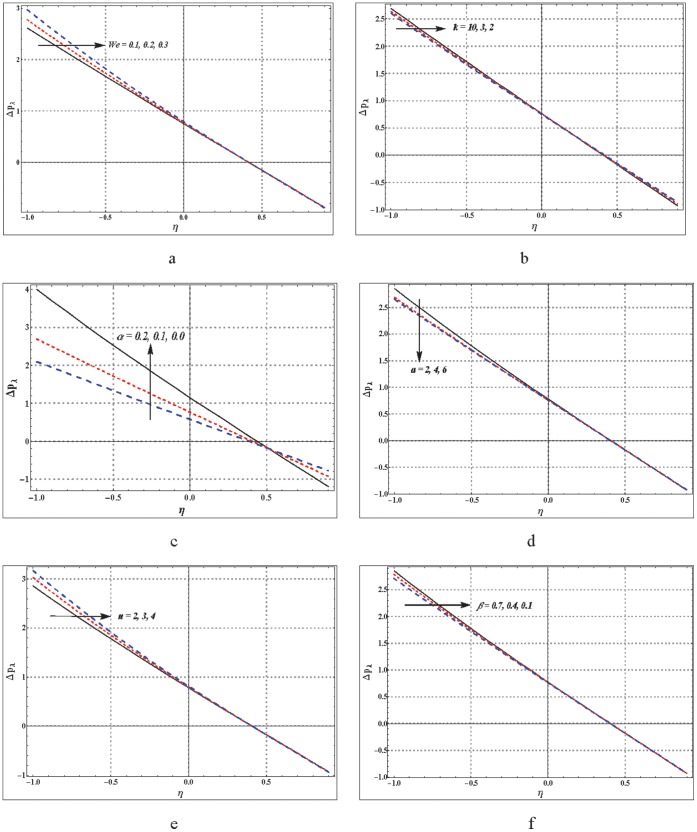
a–f. Effects of embedded parameters on pressure rise. (a) 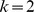, 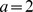, 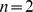, 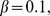 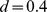, 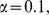 (b) 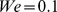, 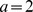, 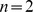, 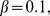 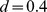, 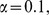 (c) 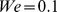, 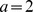, 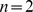, 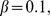 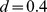, 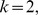 (d) 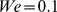, 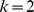, 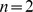, 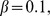 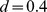, 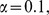 (e) 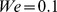, 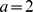, 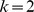, 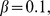 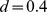, 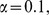 (f) 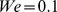, 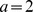, 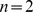, 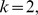 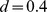, 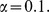.

**Figure 2:**
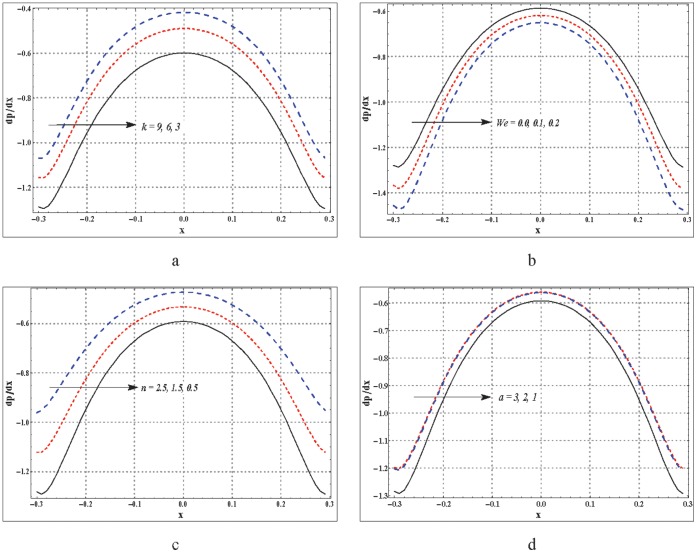
a–d. Pressure gradient for variation in different parameters. (a) 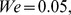 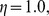 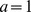, 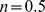, 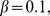 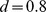, 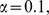 (b) 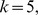 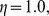 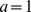, 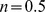, 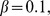 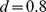, 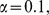 (c) 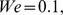 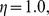 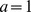, 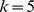, 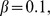 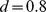, 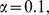 (d) 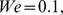 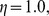 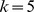, 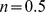, 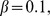 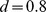, 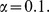.

**Figure 3:**
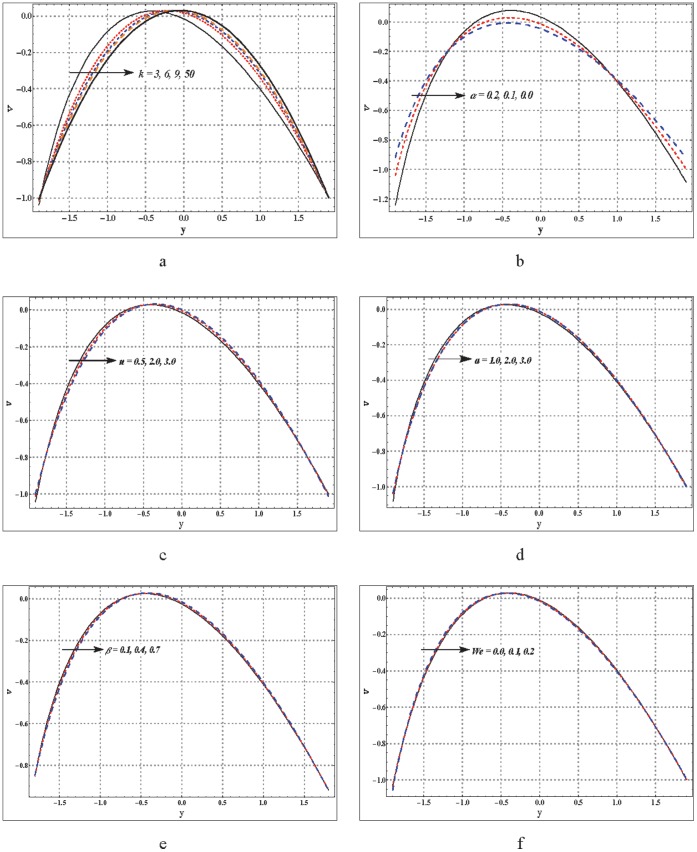
a–f. Variation of velocity profile for different embedded parameters. (a) 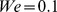, 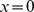, 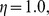 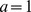, 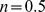, 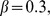 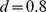, 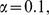 (b) 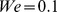, 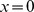, 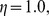 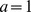, 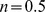, 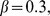 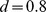, 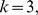 (c) 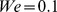, 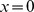, 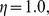 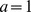, 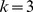, 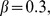 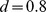, 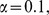 (d) 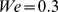, 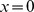, 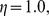 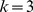, 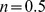, 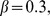 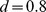, 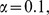 (e) 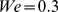, 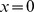, 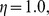 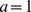, 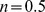, 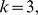 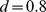, 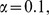 (f) 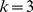, 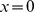, 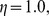 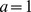, 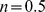, 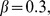 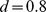, 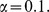.

**Figure 4:**
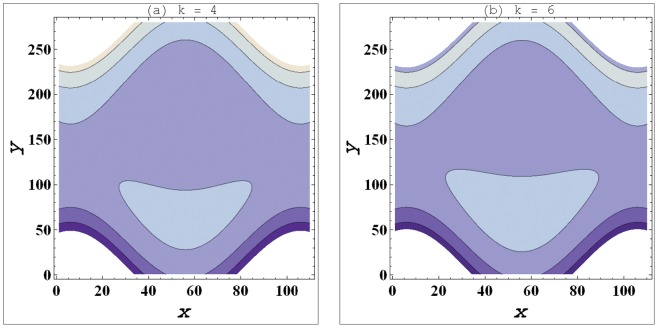
a–b. Streamlines for variation in curvature parameter (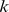) when 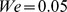, 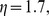 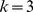, 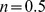, 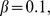 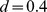, 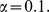.

**Figure 5:**
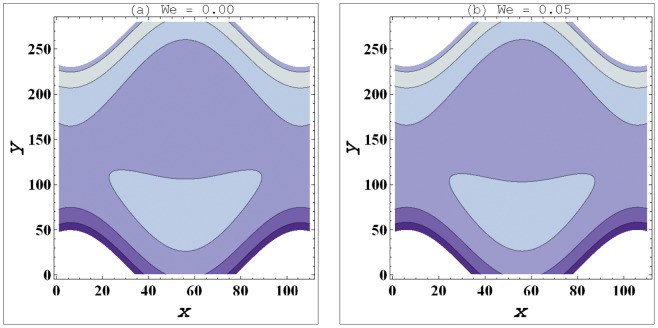
a–b. Streamlines for variation in Weissenberg number (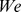) when 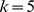, 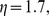 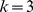, 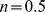, 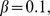 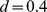, 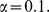.

**Figure 6:**
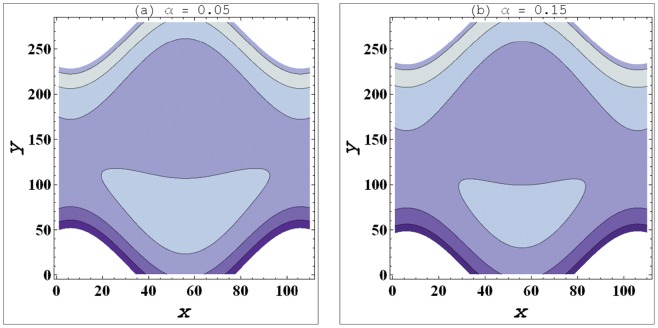
a–b. Streamlines for variation in slip parameter (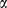) when 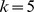, 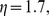 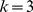, 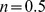, 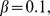 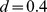, 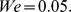.

This section is explicitly prepared to analyze the impact of various embedded parameters on the different flow quantities. Plots for pressure rise per wavelength 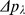, pressure gradient 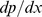, axial velocity 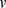 and stream function 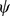 are displayed and analyzed through Figs. 1 (a–f), Figs. 2(a–d), Figs. 3 (a–f) and Figs. 4, 5, 6 respectively.

### Analysis of Pressure Rise

Pressure rise per wavelength is plotted against the flow rate in the Figs. 1 (a–f). These plots give an insight to the variation in pressure over one complete wavelength of peristaltic wave. Impact of Weissenberg number, curvature parameter, slip parameter and non-Newtonian parameters has been pointed out. Figs. 1 (a–f) depict that the pressure rise tends to decrease rapidly as the flow rate is increased. It is observed that the peristaltic pumping region 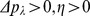 slightly increases with an increase in 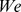 and 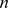 whereas opposite behavior is seen for 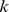, 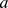 and 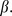 On the other hand an increase in slip parameter decreases the peristaltic pumping region by a large amount. Likewise the free pumping flux 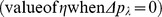 also decreases when slip effects are taken into account. The retrograde pumping region 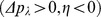 increases with increase in 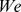 and 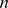 but it decreases for an increase in 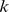, 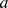
and 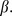 Considerably large decrease in the retrograde pumping region is noticed when the no-slip condition does not hold.

### Behavior of Pressure Gradient

Pressure gradient is plotted against the axial distance in the Figs. 2 (a–d). These Figs. showed that pressure gradient has maximum value near the wider part of peristaltic wave (near 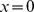) and it tends to decrease when we move towards the occlude region. Fluid flowing in a curved channel experiences a larger pressure gradient when compared for a straight channel (see Fig. 2 a). Fig. 2 b predicts that the pressure gradient in case of C-Y fluid is lesser than the viscous fluid. Increase in 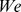 results in a decrease for pressure gradient. Increase in pressure gradient is seen corresponding to an increase in the values of 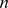 and 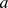 (see Figs. 2 c and d).

### Axial Velocity

Analysis of axial velocity has been carried out through Figs. 3 (a–f). These Figs. show that the velocity traces a parabolic trajectory with maximum value occurring near the center of channel. Fig. 3 a reveals the fact that the curved nature of the channel disturbs the symmetry of the velocity profile about the center of channel. The maximum value of velocity in curved channel shifts from the center towards the lower wall. As the value of curvature parameter 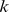 increases (as we move from curved to straight channel) then the symmetry of velocity profile about the center is retrieved. The fluid velocity in no-slip case is higher (see Fig. 3 b). Increase in 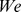 also shifts the maximum value towards the lower wall. Decrease in the velocity near the lower wall is observed subject to decrease in the values of 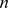, 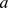 and 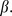.

### Streamlines Analysis

Streamlines for this problem are plotted for variation in curvature parameter 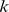, Weissenberg number 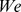 and slip parameter 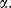 These plots have been prepared to examine the trapping phenomenon. Volume of the fluid during the flow gets trapped within a streamline. This volume of fluid is often termed as bolus. Size of such bolus is found to increase by increasing 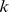 (see Figs. 4 a–b). It means that size of bolus is smaller in case of curved channel. Increase in value of velocity slip parameter decreases the size of trapped bolus. This fact indicates that the bolus has large size in no-slip situation. Figs. 5 a-b depict that *We* has very little impact on the bolus size.

## Concluding Remarks

Peristaltic motion of Carreau-Yasuda fluid in a curved channel is analyzed in the presence of slip condition. The major findings have been listed below.

- Increase in velocity slip parameter decreases the peristaltic and retrograde pumping regions.
- Free pumping flux almost remains invariant for change in 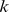, 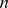, 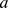, 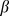 and 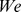.
- Pressure gradient increases for large 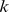, 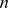 and 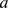 whereas it has opposite behavior for 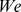.
- Symmetry of velocity profile about the center line is disturbed in case of curved channel.
- Fluid possesses higher velocity in no-slip situation.
- Size of trapped bolus is large for straight channel with no-slip at the wall.
- Effects of non-Newtonian parameters 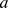, 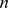, 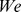 and 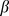 on flow quantities depend largely on each other.
